# Trisomy 1q32 and monosomy 11q25 associated with congenital heart defect: cytogenomic delineation and patient fourteen years follow-up

**DOI:** 10.1186/s13039-014-0057-8

**Published:** 2014-08-22

**Authors:** Vera Ayres Meloni, Sylvia Satomi Takeno, Ana Luiza Pilla, Claudia Berlim de Mello, Maria Isabel Melaragno, Leslie Domenici Kulikowski

**Affiliations:** 1 Department of Morphology and Genetics, Universidade Federal de São Paulo, São Paulo, SP, Brazil; 2 Children’s Interdisciplinary Neuropsychological Center, AFIP, Universidade Federal de São Paulo, São Paulo, SP, Brazil; 3 Department of Pathology, Cytogenomics Lab, LIM 03, Universidade de São Paulo, Av. Dr. Enéas de Carvalho Aguiar, 155, 2° andar, bloco 12, sala 7, São Paulo, SP, Brazil; 4 Human Reproduction and Genetics Center, Department of Collective Health, Faculdade de Medicina do ABC, Santo André, SP, Brazil

**Keywords:** Partial duplication 1q, Partial deletion 11q, Congenital heart defects, Array, FISH, Intellectual disability, Clinical follow-up

## Abstract

**Background:**

Partial duplication 1q is a rare cytogenetic anomaly frequently associated to deletion of another chromosome, making it difficult to define the precise contribution of the different specific chromosomal segments to the clinical phenotype.

**Case presentation:**

We report a clinical and cytogenomic study of a patient with multiple congenital anomalies, heart defect, neuromotordevelopment delay, intellectual disability, who presents partial trisomy 1q32 and partial monosomy 11q25 inherited from a paternal balanced translocation identified by chromosome microarray and fluorescence *in situ* hybridization.

**Conclusion:**

Compared to patients from the literature, the patient’s phenotype is more compatible to the 1q32 duplication’s clinical phenotype, although some clinical features may also be associated to the deleted segment on chromosome 11. This is the smallest 11q terminal deletion ever reported and the first association between 1q32.3 duplication and 11q25 deletion in the literature.

## Background

Partial duplication 1q is a rare cytogenetic anomaly frequently associated to deletion of another chromosome, making it difficult to define the precise contribution of the different specific chromosomal segments to the clinical phenotype. The duplicated material can result either from an inherited rearrangement or due to an unbalanced de novo translocation, duplication, or insertion [1]. The terminal duplication 1q is associated to pre and postnatal growth retardation, dysmorphisms, multiple congenital malformations, including heart defects, and intellectual disability [2]. Only 12 patients have been described in literature with duplication of the distal long arm of chromosome 1 without other concomitant chromosomal imbalance [3]-[14]. Most cases of these 1q duplication involve the 1q32qter region; and translocations involve multiple chromosomes, including 3p, 5p, 7p, 21q, and Xq [15].

The 11q terminal deletion was first described by Jacobsen et al. [16] and has a recognized pattern of malformation. Jacobsen syndrome (JBS, OMIM 147791) is a contiguous gene disorder caused by 7 to 20 Mb 11qter deletions. More than 100 cases have been described in literature and the clinical findings include developmental delay, short stature, congenital heart defects (CHD), thrombocytopenia, genitourinary anomalies, pyloric stenosis, and ophthalmologic anomalies.

Here we report on a patient with a duplication of 37 Mb of the distal long arm of chromosome1 from 1q32 to 1qter associated to a 2 Mb 11q25qter deletion inherited from his father’s balanced translocation. SNP-array and fluorescence *in situ* hybridization (FISH) techniques were used to define the specific breakpoints. The patient’s phenotype was reviewed and compared with previous patients with distal 1q duplication and distal 11q deletion.

## Case presentation

**Figure 1:**
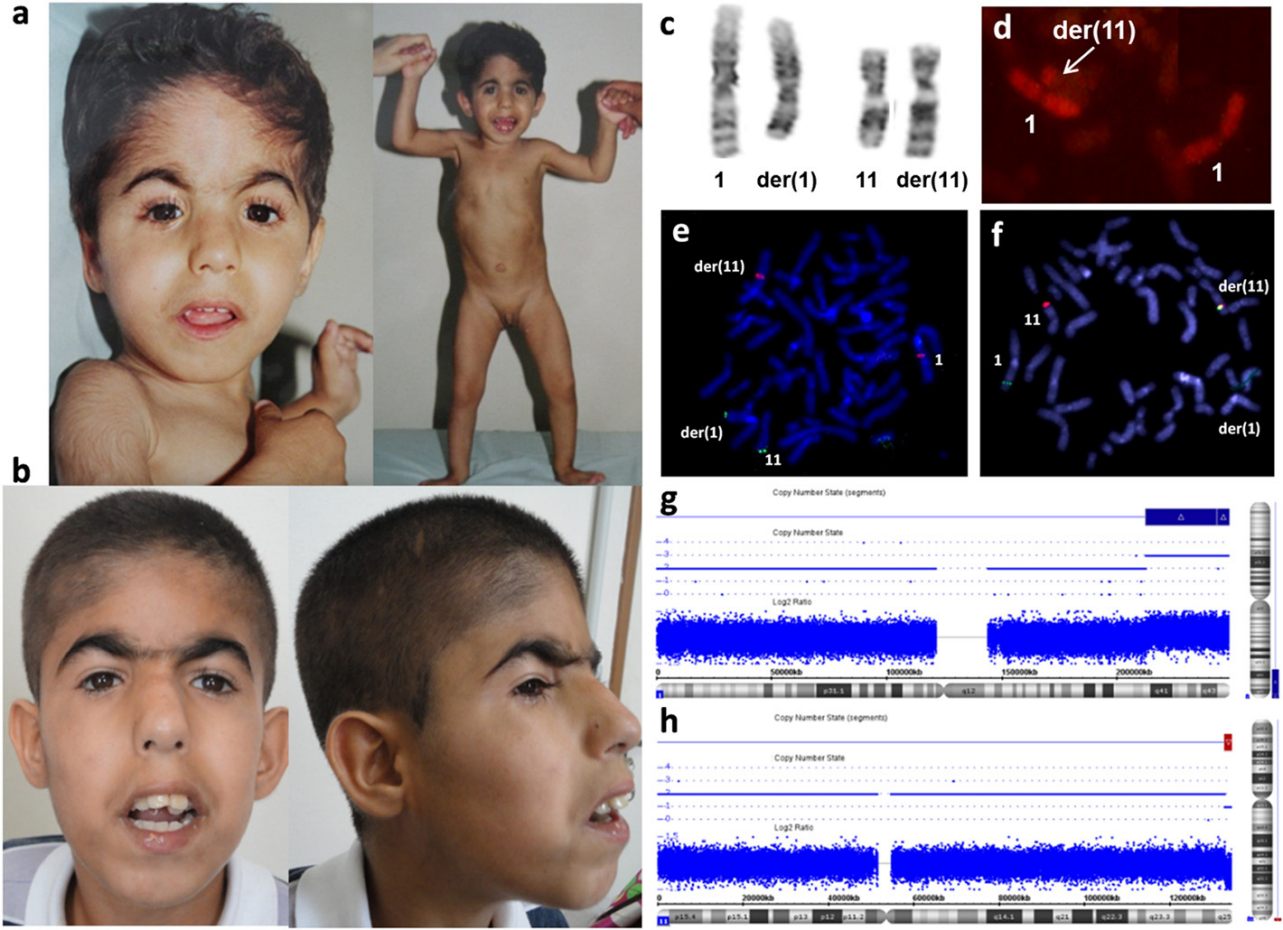
Patient at 1 year and 10 months (a) and 14 years of age (b) showing the facial dysmorphic features; Partial G-banding karyotype showing paternal balanced translocation (c); Partial FISH metaphase with WCP1 probe showing the patient’s der(11) with the duplicated segment (d); BAC-FISH results showing the breakpoint delineation in 1q32.3 and 11q25 regions in father’s metaphases (e and f); Array result for duplication (blue bar) 1q32.(212,508,954-249,224,376) × 3 (g); and deletion (red bar) 11q25(132,927,027-134,944,770) × 1 (h).

The Research Ethics Committee of UNIFESP approved this study, and consent was obtained from the patient’s parents. The male patient is the first child of a young non-consanguineous and healthy couple and has one normal sister and one paternal half-sister. He was born at term by vaginal delivery after a pregnancy with hypertension and a bleeding episode, with a birth weight of 1,980 g (10th centile), length 42 cm (10th-25th centile), and occipital frontal circumference (OFC) unavailable. He stayed 11 days in the nursery because of low birth weight and dysmorphisms. Hematologic abnormalities were not observed during the neonatal period. At the age of three months, a computerized cranial tomography showed hydrocephaly and at age of six months, the echocardiography showed pulmonary stenosis. At one year old, he had endocarditis, being hospitalized for 18 days. He also had three episodes of bronchopneumonia by the age of two years. At one year and 10 months old, he presented height of 72 cm (<5th centile), weight of 7,200 g (<5th centile), OFC of 47 cm (10th-25th centile). The clinical evaluation revealed: microsomia, relative macrocephaly, brachycephaly, downslanted palpebral fissures, long and marked eyelashes, synophrys, large ears, hipoplastic ala nasi, prominent nose, high nasal bridge, long and smooth philtrum, abnormal tooth implantation, high palate, forehead abnormal hair implantation, hirsutism, *pectus carinatum*, thoracic asymmetry, vertical palmar creases one on the left hand and two on the right, overlying position of toes, micropenis and bilateral cryptorchidism (Figure 1a). He evolved with moderate development and speech delay, and had a few convulsion episodes at age of two years. A new cardiac evaluation showed atrial septal defect, which was surgically corrected at age of 5 years. The clinical evaluation at age of ten years showed: height 119 cm (<5th centile), weight 25 kg (5th centile) and OFC 50.5 cm (50th centile). At this time, the patient additionally presented muscular hypotonia and presented with moderate intellectual disability, aggressive and hyperactive behavior, limited verbal language repertoire and dysarthria. At age of 14 years, he showed height 125 cm (<5th centile), weight 26 kg (5th centile) and OFC 52.5 cm (50th centile) (Figure 1b). At this time, the patient underwent an assessment of intellectual and neuropsychological skills and had a very limited verbal repertoire, but showed communicative intention, good eye contact and used some gestures to express his intentions. Other behavioral characteristics included mild psychomotor disabilities and lack of symbolic skills, indicating severe cognitive impairment. In daily life activities, according to the mother, he needs supervision.

Chromosome analysis from lymphocyte cultures revealed additional material in chromosome 11q. The patient’s mother had a normal karyotype but the father carries a balanced translocation t(1;11)(q32;q25) (Figure 1c). Breakpoints were defined by FISH with bacterial artificial chromosome probes (BACs). FISH analyses were performed with whole chromosome paint (WCP1-Cytocell, Cambridge, UK) and using different BAC probes from chromosomes 1 and 11, as previously reported (Figure 1d, e and f) [17]. Further investigations using Genome-Wide Human SNP 6.0 array (Affymetrix Inc., Santa Clara, CA, USA) on the patient revealed the final cytogenomic result as:

46,XY.ish der(11)t(1;11)(q32.3;q25)pat(wcp1+,RP11-663C5-,RP11-262H5+,RP11-15 J5+,RP11-265 F9-). arr[hg19]1q32.3q44(212,508,954-249,224,376) × 3,11q25 (132,927,027-134,944,770) × 1 (Figure 1g and h).

## Conclusions

This is the first report of a patient with concomitant 1q32 duplication and 11q25 deletion in the literature. The clinical features observed in our patient are more closely similar to 1q32 duplication than to 11q25 deletion. Comparing our patient with the 13 patients from literature observed by Balasubramanian et al. [14] we concluded that he presents important clinical features observed in the “Distal Trisomy 1q Syndrome”, such as marked developmental delay, microsomia, prominent forehead, downslanted palpebral fissures, long lashes, relative macrocephaly, recurrent pulmonary infections, genitourinary anomalies and CHD. However, the wide range and variety of the clinical features observed in patients with 1q32 duplication make the genotype-phenotype correlation extremely difficult.

The 1q32 duplicated segment in our patient presents 467 genes, including: *TGFB2* gene (transforming growth factor, beta 2), *RYR2* gene (ryanodine receptor 2), and *TBCE* gene (Homo sapiens tubulin folding cofactor E).The *TGFB2* gene regulates proliferation, differentiation, adhesion, migration, and other functions in many cell types and disruption of the *TGFB/SMAD* pathway, regulated by this gene, has been implicated in a variety of human cancers. The knockout of this gene in results in perinatal mortality and a wide range of developmental malformations, including CHD. The *TGFB2* gene is also involved with Loyes-Dietz syndrome 4 (OMIM **#**614816) caused by heterozygous mutation in the *TGFB2* gene. Lindsay et al. [18] identified two unrelated patients with aortic aneurysm and de novo 1q41 microdeletions encompassing the *TGFB2*, one with Marfan syndrome and the other with Loyes-Dietz syndrome-like features. The *RYR2* gene, which maps to 1q42q43, encodes a ryanodine receptor found in cardiac muscle. Mutations in this gene are associated with stress-induced polymorphic ventricular tachycardia and arrhythmogenic right ventricular dysplasia. Priori et al. [19] identified *RYR2* gene mutations in 12 patients with typical catecholaminergic polymorphic ventricular tachycardia and speculated it as a likely candidate for this genetically transmitted arrhythmic disorder. This gene is not involved in conotruncal congenital malformations as observed in our patient and other patients with 1q32 duplication. The genes *RYR2* and *VTSIP* are also associated to CHD. Other genes labeled in the 1q32.3qter duplicated segment also play an important role in neurodevelopment such as *TBCE* gene located in 1q43.2.

The 11q terminal deletion disorder (Jacobsen syndrome) presents a recognized pattern of malformation, which depends on the size of the 11qter deletion, usually ranging from 7 to 20 Mb. Since the first report in 1973 by Jacobsen et al. [16] more than 100 cases have been reported in literature. The main clinical features include motor developmental delay, pre and postnatal growth retardation, trigonocephaly, thrombocyto- or pancytopenia, cardiac defects, hypertelorism, downslanted palpebral fissures, short nose, anteverted nares, thin upper lip, V-shaped mouth, dental anomalies, and low-set malformed ears [20]. Evers et al. [21] described a 2-year-old boy with a 2.2 Mb deletion of 11q25qter and a 15.7 Mb terminal duplication of 16q22.3qter, who has no heart disease or platelet dysfunction, which are typically found in more than 90% of patients with terminal 11q deletion. This suggests that the deletion presented in their patient does not encompass the critical region for the Jacobsen syndrome. Our patient presents a smaller 2 Mb deletion than Evers et al. [21] patient, but shares some main clinical features of Jacobsen syndrome, such as cardiac congenital malformation, motor developmental delay, short stature, undescended testes, high forehead, ocular hypertelorism, downslanted palpebral fissures, anteverted nares, thick eyebrows and high-arched palate, but he has no hematologic abnormalities as observed in patients with Jacobsen syndrome. However, some of these clinical features are also described in duplication 1q32 making it extremely difficult to determine the accurate genotype-phenotype correlation. The 11q deletion observed in our patient is the smallest segment described in literature and encompasses 25 genes including the *JAM3* (junctional adhesion molecule 3) gene, a candidate gene for the Jacobsen syndrome cardiac phenotype [22] and conotruncal heart defects. This gene may be related to the atrial septal defect presented by our patient.

An early conclusion from the genotype–phenotype findings was that CHD are strongly associated with an altered dose of genes that act together. And most important, the new cytogenomic approaches enabled the detailed study of single cases and provided additional information to the genotype-phenotype correlation.

## Consent

Written informed consent was obtained from the parents of the patient for publication of this Case report and any accompanying images. A copy of the written consent is available for review by the Editor-in-Chief of this journal.

## Abbreviations

CHD: Congenital heart defects

*JAM3*: Junctional adhesion molecule 3 gene

OFC: Occipital frontal circumference

*RYR2*: Ryanodine receptor 2 gene

*TBCE*: Homo sapiens tubulin folding cofactor E

*TGFB2*: Tubulin folding cofactor E gene

*VTSIP*: Ventricular tachycardia, stress-induced polymorphic gene

## Competing interests

The authors declare that they have no competing interests.

## Authors’ contributions

MIM and LDK coordinated and participated in the design of the study. VAM, ALP and CBM carried out the clinical assessments. SST carried out chromosomal classic and FISH analysis and LDK carried out microarray analysis. VAM drafted the manuscript and all authors read and approved the final manuscript. All authors read and approved the final manuscript.
